# Microwave catalyzed carbothermic reduction of zinc oxide and zinc ferrite: effect of microwave energy on the reaction activation energy†

**DOI:** 10.1039/d0ra04574h

**Published:** 2020-06-23

**Authors:** Mamdouh Omran, Timo Fabritius, Eetu-Pekka Heikkinen, Tero Vuolio, Yaowei Yu, Guo Chen, Yilmaz Kacar

**Affiliations:** Process Metallurgy Research Group, Faculty of Technology, University of Oulu Oulu Finland mamdouh.omran@oulu.fi; State Key Laboratory of Advanced Special Steel, School of Materials Science and Engineering, Shanghai University Shanghai China; Kunming Key Laboratory of Energy Materials Chemistry, Yunnan Minzu University Kunming China guochen@kust.edu.cn; Material Science and Engineering Dep., Carnegie Mellon University Pittsburgh USA

## Abstract

Recently, more attention has been paid to the use of microwave (MW) energy in accelerating chemical reactions. The effect of microwave energy on the reduction of zinc oxide and zinc ferrite was investigated. The results indicated that the temperatures required to initiate zinc oxide and zinc ferrite reduction under MW heating were 550 and 450 °C, respectively, while under conventional thermal (CT) heating, were 950 and 850 °C, respectively. Apparently, the MW reaction had a negative standard Gibbs free energy (Δ*G*) at a lower temperature (∼400 °C) when compared to the CT reaction. Additionally, the activation energy (*E*_a_) substantially decreased from 223.7 and 221.1 kJ mol^−1^ under CT heating to 64.8 and 32.9 kJ mol^−1^ under MW heating for Zn oxide and zinc ferrite, respectively. The enhancement in zinc reduction under MW energy was due to the rapid and bulk heating phenomena of MWs as well as the interactions occurring between the electromagnetic MW pattern and the molecules of heated materials.

## Abstract 2

Catalytic effect of microwave energy on carbothermic reduction of zinc ferrite.

## Graphical abstract

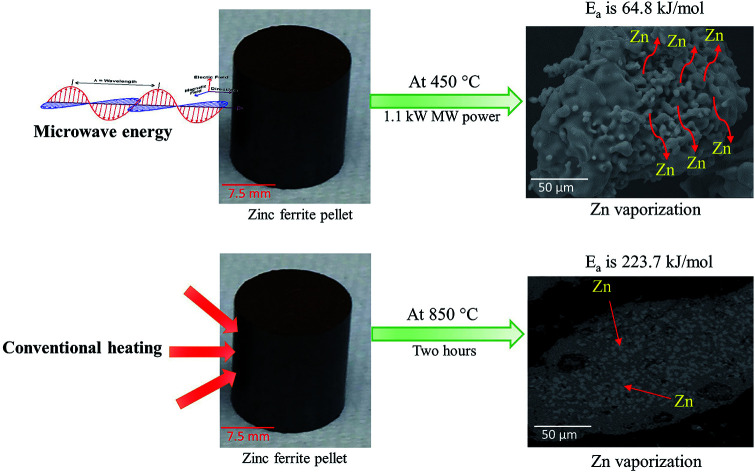

## 1. Introduction

Recently, attention in different fields of chemistry and metallurgy has been increasingly paid to using microwave (MW) energy to increase the reaction rates and improve the recovery of valuable metals. Recent studies have shown that the use of MW energy results in a substantial improvement in the rate of reaction when compared to conventional thermal heating.^1–3^ Microwave energy is a novel type of electromagnetic wave, with 300 MHz to 300 GHz frequencies.^4,5^ Microwave heating is generated by interaction between the dielectric material and the MW field.^6,7^ This contrasts with conventional thermal heating, which heats the sample from the outside-in through heat transfer mechanisms.^8,9^

The reasons behind the MW enhanced reaction rates are still unclear and speculative. Huang and Yang^1^ related these enhancements to the rapid and bulk heating phenomena of MW as well as the interactions occurring between the electromagnetic MW pattern and molecules of heated materials. Researchers investigated that increasing the rate of chemical reactions under microwave reaction is owing to reduce the activation energy that is needed to activate the reactant molecules.^2,3^ They concluded that the microwave heating mechanism affects the thermodynamics of the reaction, which resulted in energy saving.

An increasing amount of attention has been paid to microwave energy in several fields in terms of mineral processing, material preparation, and environmental applications.^10–14^ There is a paucity of information on the inherent mechanism of the electromagnetic wave effect on the chemical reactions, and insufficient work have focused on decreasing the reaction activation energy under microwave energy. The thermodynamics of the microwave reduction of zinc ferrite has been studied by Wang *et al.*^15^ Saidi and Azari^16^ studied the carbothermic reduction of zinc oxide using microwave. However, the nature of MW's effect on carbothermic reduction has not yet been discussed.

The objective of this work is to investigate the catalytic effect of microwave energy on the carbothermic reduction of zinc oxide and ferrite. In addition, activation energy was calculated to confirm the effect of MW energy on the reduction reaction in comparison to conventional thermal reduction.

## 2. Experimental

### 2.1. Materials preparation

Reagent-grade chemicals zinc oxide, iron(iii) oxide, and synthetic graphite from Alfa Aesar with purities >99.0% and −325 mesh particle sizes were used. The zinc ferrite was prepared from reagent-grade zinc oxide and iron(iii) oxide with a molar ratio of 1 : 1. The mixture was pressed and sintered at 750 °C for 4 h. After sintering, the tablet was ground, remixed, and repressed into a tablet again. This procedure was repeated four times to obtain the best purification and crystallization results.

After drying the samples, mixtures of zinc oxide, zinc ferrite and graphite were well homogenized using a gate and mixer. The particle size of the materials was −325 mesh. The percentage of graphite was 1.2 the stoichiometric quantity of carbon needed to reduce both zincite and zinc ferrite into elemental zinc and iron metal. The sample mass was 3 g, and 0.5% of bentonite was added to the mixture as a binder. The sample was compressed at 150 kg f cm^−2^ for 60 s by using a hydraulic oil press.

### 2.2. Conventional furnace

**Fig. 1:**
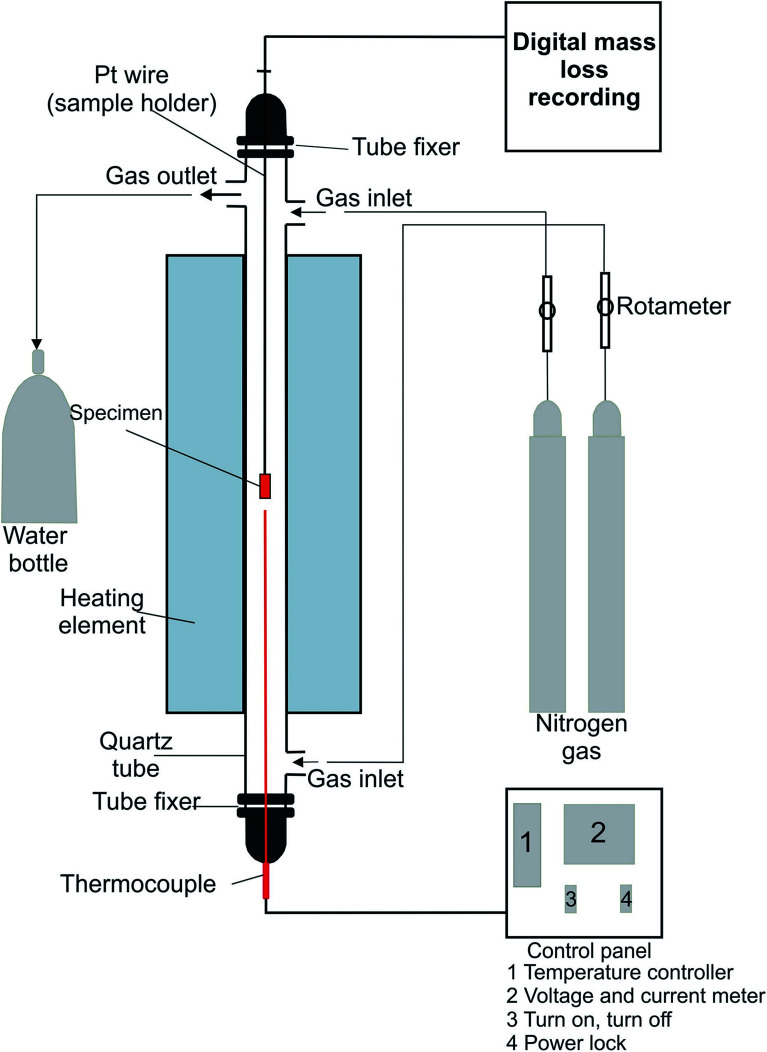
Conventional furnace experimental set-up.

A tube furnace device was used in this study. The experimental setup diagram is shown in Fig. 1.

The reduction experiments were done at 850, 950, and 1050 °C for a two-hour reaction time. Time and temperature were set from the control panel. Thermocouple was used to record the temperature. The thermocouple connected to the control panel to control the furnace temperature during the experiment. The sample was placed in a basket and inserted into the furnace through a platinum wire. The sample wire was connected to digital mass loss recording software, which measured the sample loss during the experiments.

Nitrogen gas was used to offer an inert atmosphere inside the furnace. The N_2_ flow rate from the bottom to the top was 5 L min^−1^ and 1 L min^−1^ from top to bottom. The output vapors and gases from the furnace were cooled and condensed with water. After the experiment, the sample was kept on the furnace to cool down in a N_2_ atmosphere. Then, the sample was prepared for later tests.

### 2.3. Microwave furnace

**Fig. 2:**
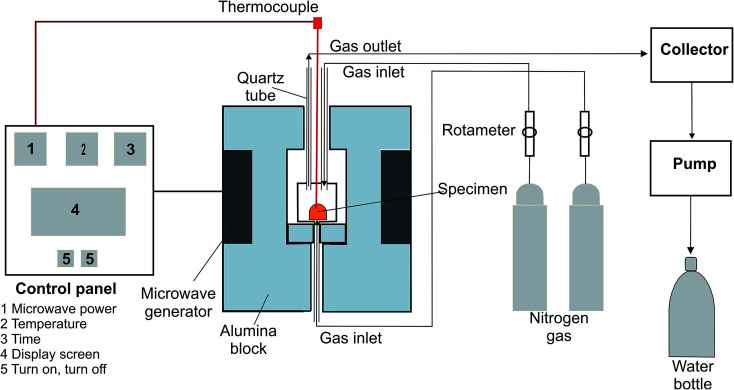
The microwave experimental set-up.

The representation of the microwave experimental arrangement is shown in Fig. 2. The microwave generator consisted of two magnetrons with 1.1 kW power and a 2.45 GHz frequency.

The reduction experiments were carried out at 450, 550, 650, 750, and 850 °C. Temperature was measured using a thermocouple. The thermocouple connected to the setting panel which regulate the temperature during microwave experiments. The sample was placed in an alumina crucible. N_2_ gas was inserted into the reaction zone from the top and bottom with the same flow rate used in the conventional furnace. The evaporated zinc vapor from the sample was drawn by a pump and stuck in a collector, and the gasses were cooled with water. The sample mass loss was measured by weighing the sample every 60 s.

After the experiment, the sample was left to cool down in the microwave furnace. Then, the sample was prepared for later analysis.

### 2.4. Characterization methods

The atomic absorption spectrometer (AAS) was applied to determine the concentration of zinc in the sample. HNO_3_ acid was used for zinc dissolving and diluted to 100× dilution, and Perkin Elmer AAnalyst 400 flame was used for analysis. The phase transformation of zinc ferrite was determined using X-ray diffraction (Rigaku SmartLab 9 kW XRD). The measurements were taken using CoKα radiation operated at 40 kV voltage and a 135 mA (5.4 kW) rotating anode. A scanning electron microscope (FESEM), connected with an EDS (Energy-Dispersive X-ray Spectroscopy) analyzer (Zeiss ULTRA plus FESEM), was used to investigate the micromorphology and microanalyses of the samples.

Thermogravimetry (TG) – differential scanning calorimetry (DSC) were carried out using a Netzsch STA 409 PC Luxx. The tests were performed in an air and nitrogen atmosphere conditions from 20 to 1300 °C and a heating rate of 10 °C min^−1^. Around 30.84 mg of the material was placed in a platinum crucible.

## 3. Thermodynamic calculation

Thermodynamic calculations were executed using FactSage (version 7.2) and its FactPS, FToxid, and FSstel databases. These calculations were made to estimate the occurrence of zinc in different phases as a function of temperature. The calculations were made for 100 grams of material at a total pressure of 1 atm, whereas the initial amount of carbon was calculated separately for each case. The amount of carbon was chosen to be 1.2 times the amount needed to reduce all the zinc and iron into an elemental form, according to the reactions presented in eqn (1)–(3):

1 ZnO_(s)_ + C_(s)_ = Zn_(g)_ + CO_(g)_

2 ZnFe_2_O_4(s)_ + 4C_(s)_ = Zn_(g)_ + 2Fe_(s)_ + 4CO_(g)_

3 Fe_2_O_3(s)_ + 3C_(s)_ = 2Fe_(s)_ + 3CO_(g)_

**Fig. 3:**
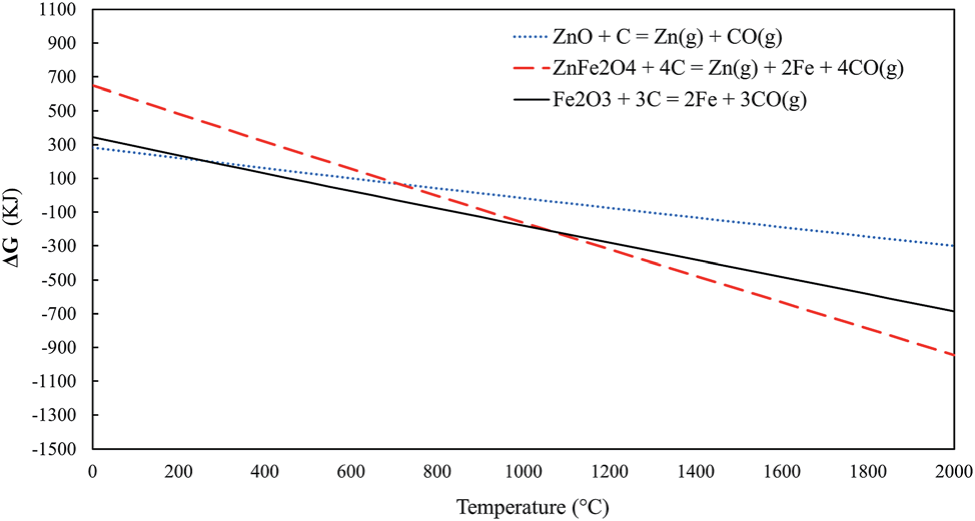
Standard Gibbs free energies for eqn (1)–(3).

Standard Gibbs free energies for eqn (1)–(3) are presented in Fig. 3. The reduction of zinc oxide and zinc ferrite into zinc becomes spontaneous at around 950 °C and 800 °C, respectively. On the other hand, the reduction of hematite becomes spontaneous at approximately 650 °C.

**Fig. 4:**
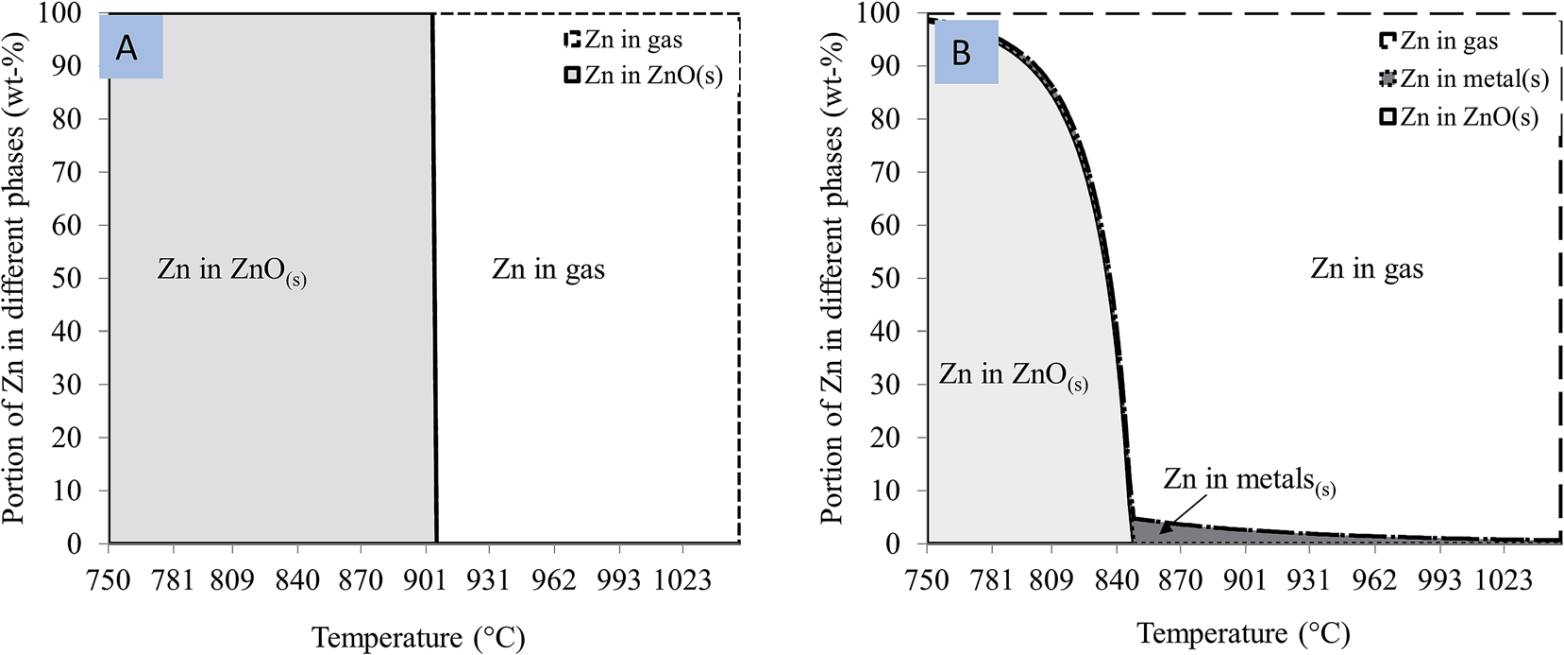
Portion of zinc in different phases of thermodynamic equilibrium as a function of temperature when (A) zinc oxide and (B) zinc ferrite is heated with carbon.

The equilibrium distributions of zinc in the different phases of the thermodynamic calculations are shown in Fig. 4. These diagrams provide an idea of how much zinc it is theoretically possible to reduce in different cases and at different temperatures.

It can be seen from Fig. 4A that the temperature required to reduce solid ZnO into gaseous Zn could be as high as 900 °C and takes place at one temperature, after which all the zinc is in a gaseous form. Meanwhile, Fig. 4B showed that the reduction of zinc from zinc ferrite begins at lower temperatures when compared to its reduction from zincite (Fig. 4A). The disintegration of zinc ferrite occurs at temperatures above 800 °C. At this temperature, zinc ferrite was decomposed to zinc oxide and iron oxide. The equilibrium amount of ZnO decreased up to 850 °C, as zinc oxide is reduced to elemental zinc gas. Metallic iron is also formed, since the iron in zinc ferrite is reduced. According to the calculations, some zinc was dissolved in the metal phase; hence it was not possible to reduce all the zinc into the gas phase, although the portion of zinc remaining in the solid materials was very small.

## 4. Results and discussion

### 4.1. TG–DSC

**Fig. 5:**
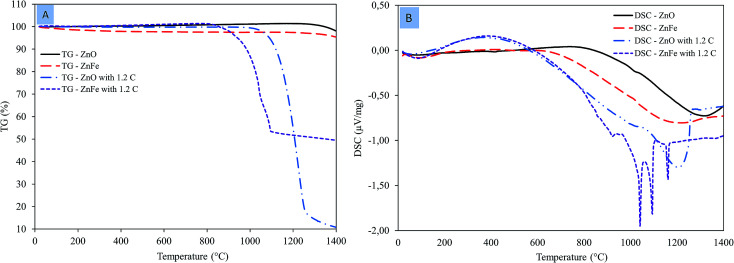
Thermoanalyses of zinc oxide and zinc ferrite. (A) TG; (B) DSC.

The TG and DSC curves of zinc oxide and zinc ferrite are revealed in Fig. 5A and Fig. 5B, respectively. The TG–DSC curves for both zinc oxide and zinc ferrite as a function of temperature under air atmosphere did not detect any mass loss or thermal reaction. At a high temperature of about ∼1200 °C, there is a deep slop in the DSC curve owing to the sintering of both zinc oxide and zinc ferrite (Fig. 5B).

TG–DSC curves for zinc oxide with 1.2 times the stoichiometric amount of carbon under nitrogen atmosphere are shown in Fig. 5. DSC curve showed that the ZnO reduction is initiated at a temperature of 967 °C.^17^ The TG curve shows that the mass loss starts at 967 °C and continues until 1265 °C owing to zinc evaporation. TG–DSC curves for zinc ferrite with 1.2 times the stoichiometric amount of carbon under nitrogen atmosphere are shown in Fig. 5. The DSC curve shows that the decomposition of zinc ferrite begins at 861 °C.^18^ A series of reactions at 861, 925, 1040, 1093, and 1159 °C observed in the DSC associated with mass loss in the TG within the same temperature range. ZnFe_2_O_4_ first decomposed to ZnO and Fe_2_O_3_ within the temperature range of 861 and 925 °C.^19^ Then the reduction of zinc oxide and iron oxide happens. The temperature required to initiate ZnO and ZnFe_2_O_4_ reduction in DSC are consistent with the thermodynamic calculations.

### 4.2. Dielectric properties and materials heating properties

The dielectric properties of both zinc oxide and zinc ferrite have been studied in detailed in previous work.^18^ The detail information is in ESI (Section I).†

Omran *et al.*, (2017) studied the effect of temperature on the dielectric constant (*ε*′) and loss factor (*ε*′′) of zinc oxide and zinc ferrite (Fig. S1, ESI†). They found that the changes in *ε*′ with temperature were insignificant, while the changes in *ε*′′ are more important at higher temperatures. The increase in *ε*′′ can be attributed to an increase in electrical conductivity owing to sample sintering.

**Fig. 6:**
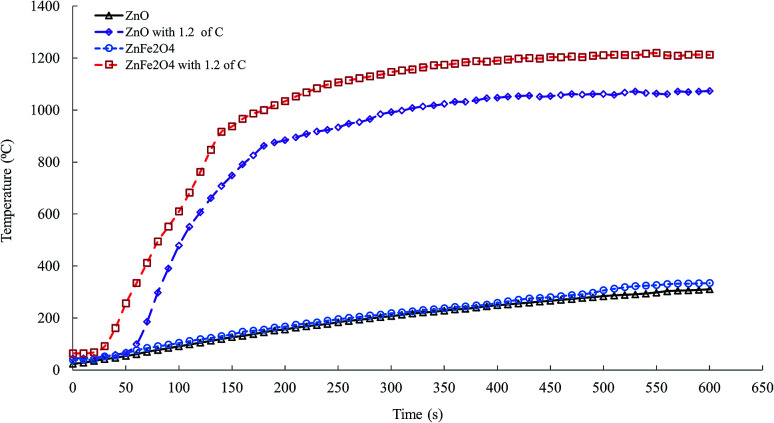
Microwave heating profile at 1.1 kW power intensity for zinc oxide and zinc ferrite.

The microwave heating profile of ZnO and ZnFe_2_O_4_ at 1.1 kW microwave power is shown in Fig. 6. After 600 s of microwave irradiation, the temperatures of ZnO and ZnFe_2_O_4_ did not exceed 310 and 334 °C, respectively. The poor microwave absorbing of ZnO and ZnFe_2_O_4_ are owing to their low dielectric properties.^20^Fig. 6 shows that the adding of graphite to ZnO and ZnFe_2_O_4_ significantly increase the microwave heating rate to high temperatures. For instance, after 180 s of microwave heating, the temperatures of ZnO and ZnFe_2_O_4_ improved from 112 and 123 °C to approximately 1000 and 863 °C after the samples were mixed with graphite.

### 4.3. Isothermal reduction experiments

For comparison, the experiments were carried out using both microwave (MW) heating and conventional thermal (CT) heating under identical conditions.

The sample mass loss during the carbothermic reduction of zinc oxide from the MW and CT reactions as a function of temperature is in (Fig. S2, ESI†). Under MW, we observed a rapid mass loss in the early stages of the reaction; however, after approximately 11 min, the mass became constant. In the case of the MW reaction, a mass loss of about 11.87 wt% was obtained at 550 °C, indicating the reduction of ZnO initiated at this temperature. Under the CT reaction, the sample mass was almost unchanged at 850 °C. By increasing the temperature to 950 °C, a mass loss of 11.63 wt% was observed, indicating that the reduction of ZnO initiated at this temperature, which was consistent with the thermodynamic equilibrium calculations.

**Fig. 7:**
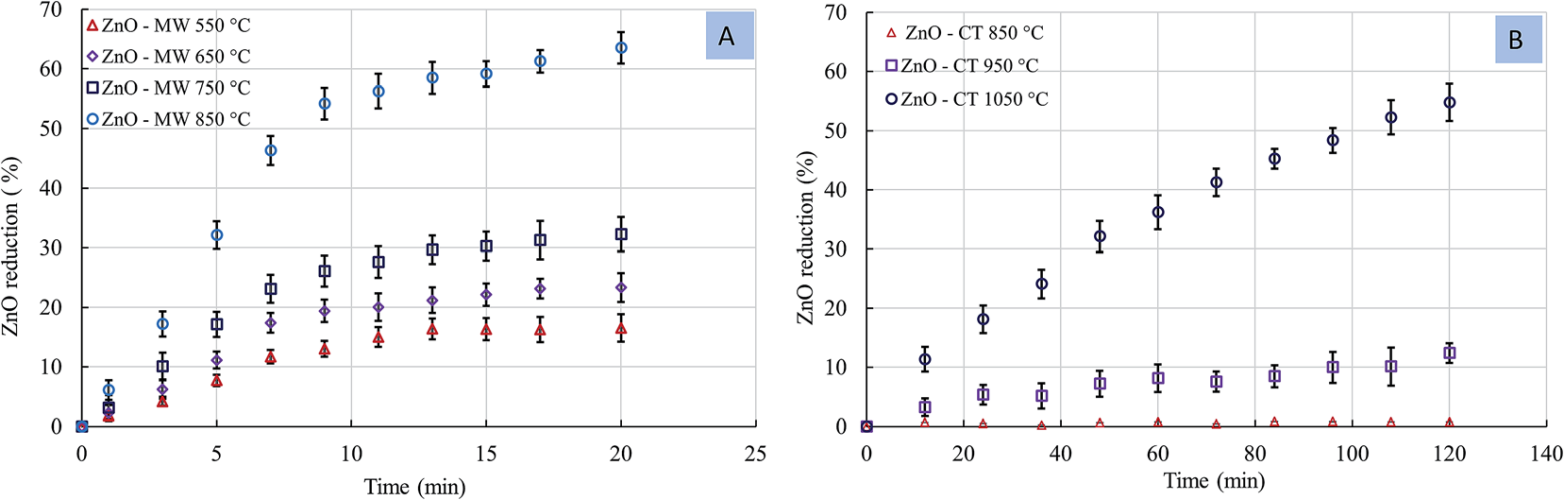
Zinc oxide reduction. (A) MW reaction; (B) CT reaction.

Fig. 7 shows the rate of ZnO reduction as a function of temperature under the MW and CT reactions. In comparing the two processes, the 63.36% zinc reduction rate was obtained at 850 °C under the MW reaction, while the 54.36% zinc reduction was obtained at 1050 °C under the CT reaction. Under the MW reaction, the ZnO reduction rate was rapid in the early stage of the reaction, while under the CT reaction, the reduction rate was linear with time. The difference in the Zn reduction rate between the MW and CF reactions was more pronounced at lower temperatures. In addition, the temperature required to initiate ZnO reduction under the MW was significantly less than that under the CT reaction. In the case of the MW, the ZnO reduction initiated at 550 °C, while a temperature of 950 °C was required to initiate the ZnO reduction in the case of CT. The difference in the reaction temperatures to initiate the ZnO reduction was ∼400 °C.

The sample mass loss of zinc ferrite under the MW and CT reactions as a function of temperature is in (Fig. S3, ESI†). We observed a rapid mass loss in the early stages of the reaction; after that, the sample mass became almost constant. In the case of the CT reaction, we did not observe any mass loss in temperature lower than 850 °C. About ∼3 wt% mass loss was observed at 850 °C, revealing that zinc ferrite decomposition was initiated at this temperature. By increasing the temperature, the mas loss increased. Meanwhile, in the case of the MW reaction, a mass loss of ∼9.91 wt% obtained at 450 °C indicated that zinc ferrite decomposed at about 450 °C.

**Fig. 8:**
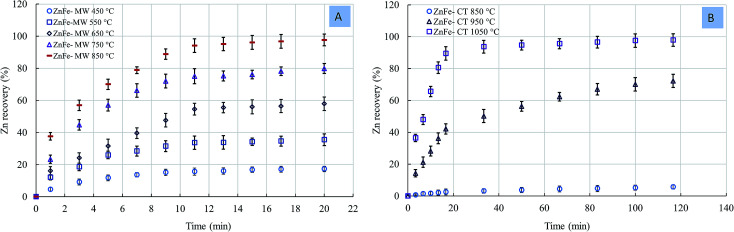
Zinc recovery rate. (A) MW reaction; (B) CT reaction.

Fig. 8 shows the rate of Zn recovery as a function of temperature. A zinc recovery rate of 17.13% was obtained with the MW at 450 °C, while 5.22% zinc recovery was obtained at 850 °C with the CT. We did not observe any reactions at temperatures lower than 850 °C in the case of the CT reaction. The temperatures required to reach ∼98% zinc recovery under the MW and CF reactions were 850 °C and 1050 °C, respectively. The difference in the Zn recovery rate between MW and CF reaction was more pronounced at lower temperatures.

**Fig. 9:**
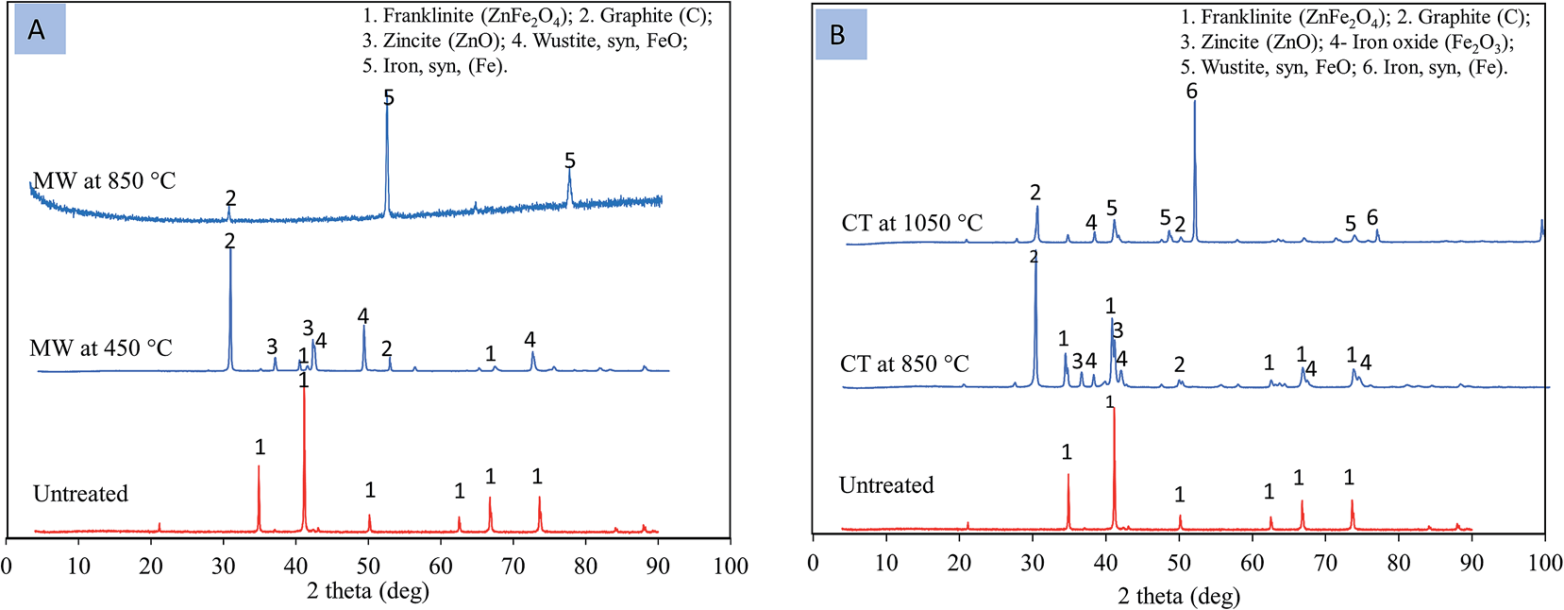
XRD patterns of zinc ferrite. (A) MW reaction; (B) CT reaction.

The XRD shows that, in the case of the MW reaction, zinc ferrite was decomposed to ZnO and FeO at a temperature of 450 °C (Fig. 9A). Meanwhile, in the case of the CT reaction, zinc ferrite was decomposed to ZnO and Fe_2_O_3_ at 850 °C (Fig. 9B). The temperature required to decomposed zinc ferrite was 400 °C lower than that of the MW reaction. The formation of FeO and ZnO with the MW instead of Fe_2_O_3_ and ZnO with the CT was due to the fast microwave reaction and the reduction of Fe_2_O_3_ into FeO in a short amount of time.

**Fig. 10:**
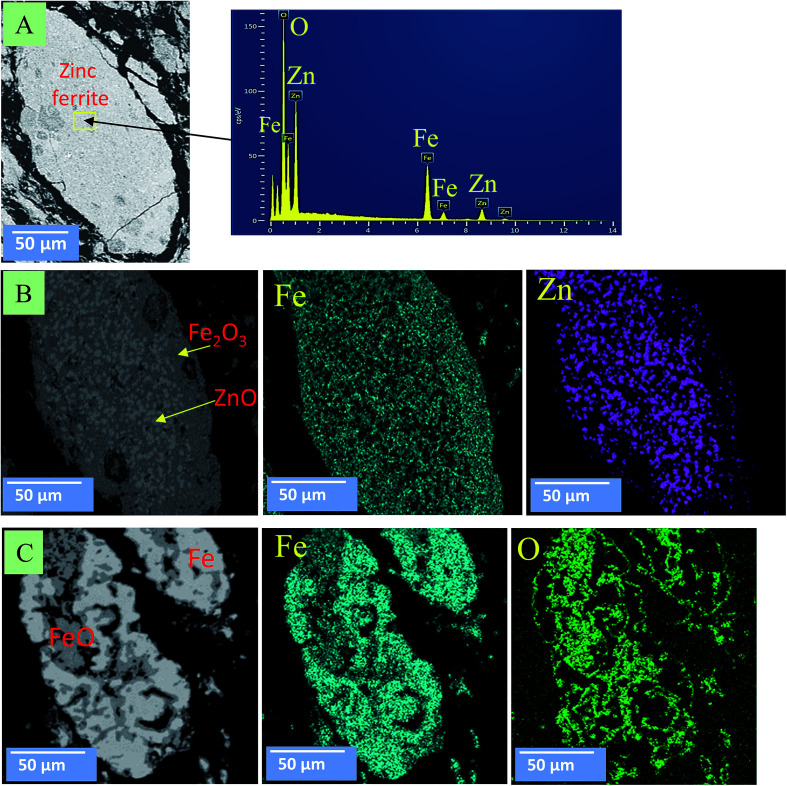
SEM images for zinc ferrite under the CT reaction. (A) At 850 °C; (B) at 950 °C; (C) at 1050 °C.

**Fig. 11:**
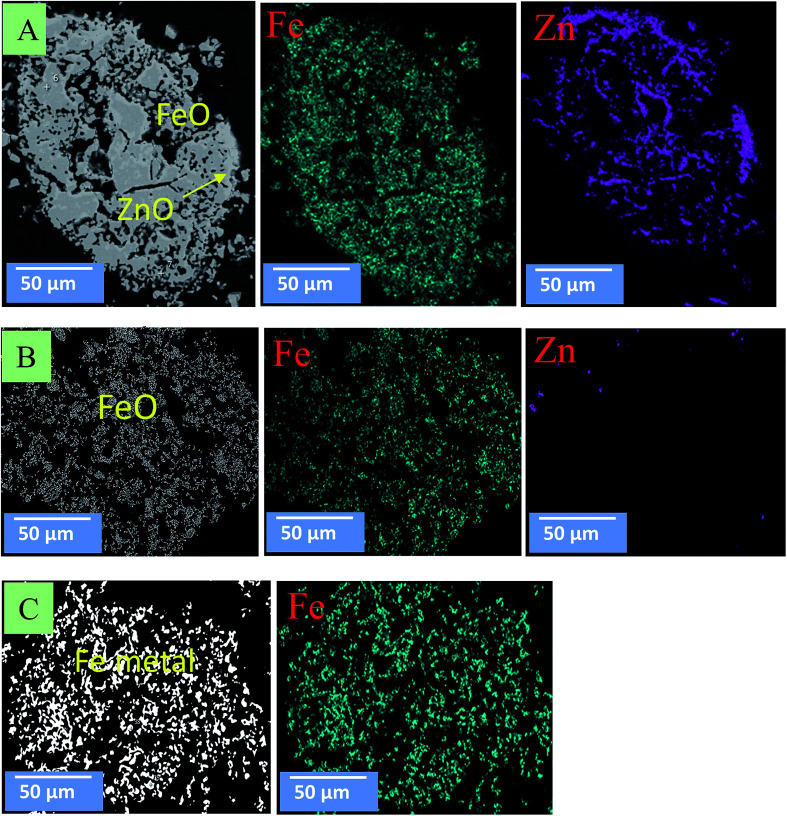
SEM images for zinc ferrite under the MW reaction. (A) At 550 °C; (B) at 750 °C; (C) at 850 °C.

Fig. 10 and 11 show the morphology changes of zinc ferrite after the MW and CT reactions. In the case of the CT reaction, no obvious change was observed at 850 °C. At 950 °C, ZnFe_2_O_4_ decomposed into ZnO and Fe_2_O_3_ phases, and ZnO reduction began on the surface. At 1050 °C, ZnO was completely removed, and Fe_2_O_3_ was reduced to FeO and Fe metal (Fig. 10). SEM images show the reduction began from the outside-inward.

In the case of the MW, the reduction reaction was rapid and volumetric. Fig. 11 shows that, at 550 °C, zinc ferrite was decomposed to ZnO and FeO. At 750 °C, ZnO reduced and continued to be removed from the sample. At 850 °C, ZnO was completely removed, and FeO was reduced into Fe metal. SEM images show that the reduction occurred in the bulk sample.

The differences in the rate of ZnO reduction and recovery specify that the influence of microwave energy on a reaction extends far beyond simple selective heating and may display different thermodynamics. Moreover, under microwave reaction, the thermodynamics of the reaction changed significantly. The microwave reaction had standard Gibbs free energies at lower temperatures when compared to the thermal reaction, as indicated in the thermodynamics section. The results indicated that the MW reaction had −Δ*G* values ∼400 °C lower than those of the CT reaction.

Some researchers^2,3,21,22^ have stated that improvements in the MW reaction rate owing to the rapid and bulk heating reaction of electromagnetic field, in addition to the interactions between the microwave electromagnetic field and the reactant molecules (non-thermal microwave effects). Zhou *et al.*^3^ revealed that MW energy improve the rate of reaction through decreasing the activation energy required to activate the reactant molecules.

### 4.4. Kinetic analysis of the isothermal reduction experiments

To estimate the reduction rate parameters, a kinetic analysis was carried out for the studied isothermal cases. The sample mass loss was attributed to the loss volatilization of Zn_(g)_ and CO_(g)_, and the reduction extent was estimated based on the reaction mass balances. The temperature dependency of the rate constant is described using Arrhenius' equation (eqn (4)):4
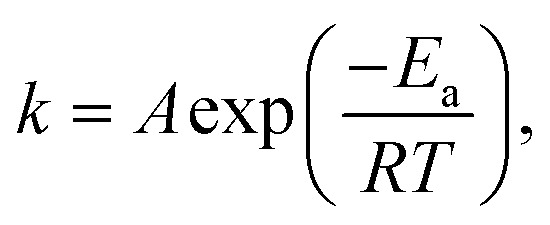
where *k* is the reaction rate constant, *A* is the pre-exponential factor, *E*_a_ is the activation energy, *R* is the molar gas constant, and *T* is the temperature. From the logistic transformation of Arrhenius' law, the rate parameters could be estimated using the least-squares method.

The rate constants for the ZnO–C system were estimated by making use of the model fitting method. It was found that the best fit was obtained by assuming the reduction could be described with the contracting sphere model, for which the integral form is eqn (5):^23^

5 *g*(*X*) = 1 − (1 − *X*)^1/3^,

where *g*(*X*) is the integral form of the conversion dependent function *f*(*X*) and *X* is the extent of conversion. An analysis of the activation energy *via* the model fitting method was carried out for the data obtained from the experiments in the conventional thermal (CT) furnace. Due to the relatively large noise in the temperatures of 850 °C and 950 °C and the small mass rate of change, the isothermal data was treated with outlier detection and a removal procedure. The outliers were detected and removed by making use of the following conditions:
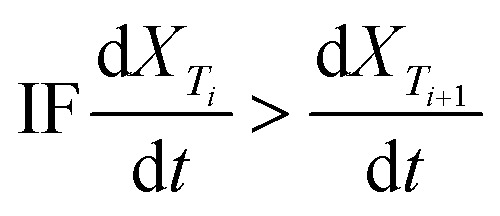
and *T*_*i*+1_ > *T*_*i*_ then remove a data point.

**Fig. 12:**
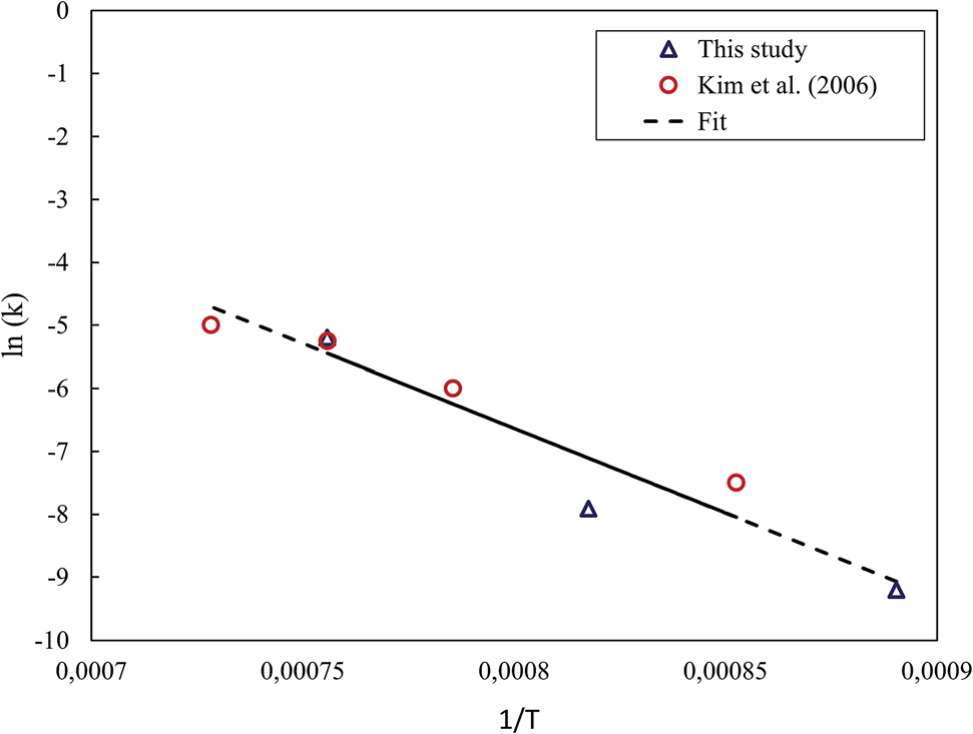
Arrhenius' plot for ZnO reduction under the CT reaction.

The removed datapoint was replaced with a polynomial least-squares estimate that was fit based on the rest of the points. Fig. 12 presents the Arrhenius' plot determined for the ZnO–C system. For the estimation of the activation energy, the data obtained in this study was combined with the data of Kim *et al.*,^24^ for which the rate constants seemed to be in reasonable agreement. Based on these, the activation energy for the reduction of ZnO under the CT reaction was estimated to be 223.7 kJ mol^−1^.

The estimation of the kinetic parameters for the reduction of ZnFe_2_O_4_ was carried out using the differential model-free method introduced in more detail in Vyazovkin *et al.*^25^ In this method, the Arrhenius' law is derived into the following form (enq (6)):6
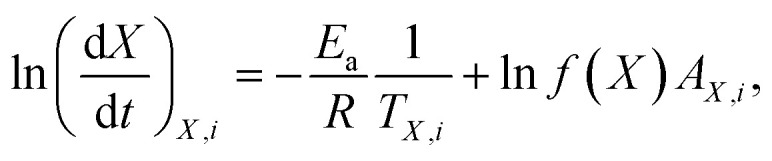
where *f*(*X*) is the differential form of the rate law. *A* property of this method is that the kinetic parameters are not dependent on the assumptions made while choosing a proper *f*(*X*) and, consequently, *g*(*X*). In this method, the activation energy is fitted for each extent of conversion, hence the method being isoconversional. However, in this case, the extent of conversion at the temperatures of 850 °C and 950 °C was very low, which made the conversion dependency of the activation energy non-observable for the data. For this reason, the activation energy was evaluated for each time instant, and the yielded vector of the activation energies was averaged. This procedure yielded an activation energy of 222.1 kJ mol^−1^ for the ZnFe_2_O_4_ system.

For the MW reaction, the analysis was carried out using the model fitting method due to the limited number of data. The best fits for both systems were obtained with the 3-dimensional diffusion equation (eqn (7)), in which the integral form is given by Vyazovkin *et al.*:^25^

**Fig. 13:**
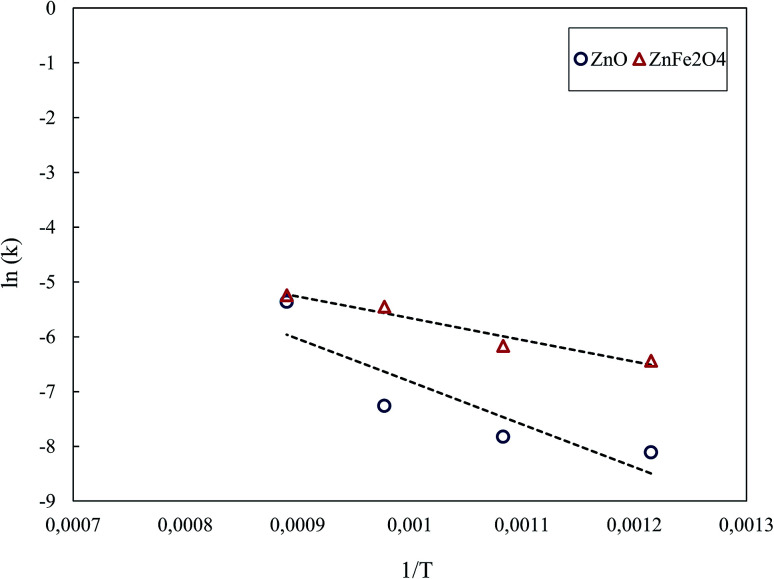
Arrhenius' plot for ZnO and ZnFe_2_O_4_ reduction under the MW reaction.

7 *g*(*X*) = (1 − (1 − *X*)^1/3^)^2^,

The Arrhenius' plot for the reduction of ZnO and ZnFe_2_O_4_ is shown in Fig. 13. The least-squares fit gave the activation energies of 64.8 kJ mol^−1^ and 32.9 kJ mol^−1^ for the reduction of ZnO and ZnFe_2_O_4_, respectively, under the MW reaction. By this, it is evident that the reduction of the zinc compounds in MW was significantly faster than that in CT.

The *E*_a_ decreased from 223.7 and 221.1 kJ mol^−1^ under the CT reaction to 64.8 and 32.9 kJ mol^−1^ under the MW reaction for ZnO and ZnFe_2_O_4_, respectively. The decrease in *E*_a_ under the MW reaction revealed that MW energy had a significant catalytic effect.^1,3^ Hunt *et al.*^2^ stated that the *E*_a_ for the carbon–carbon dioxide reaction reduced from 118.4 kJ mol^−1^ under conventional heating to 38.5 kJ mol^−1^ under MW heating. The authors concluded that MW energy can speed up the chemical reactions.

The mechanism of microwave heating, which results from the polarization of a dipole, is different from the mechanism of conventional heating, which leads to a change in the internal energy level of the molecules to reach their activated state. However, a more detailed analysis of these mechanisms is needed to be able to comprehensively describe the reasoning behind the phenomena.

## 5. Conclusions

This work investigates the catalytic effect of microwave energy on the carbothermic reduction of zinc oxide and zinc ferrite. The activation energy *E*_a_ for zinc oxide and zinc ferrite under the MW and CT reactions was calculated.

Under the MW reaction, the thermodynamics of the zinc oxide and zinc ferrite reaction greatly changed. The MW reaction had a negative Δ*G* at lower temperatures (<∼400 °C) when compared to the CT reaction. The activation energy significantly reduced from 223.7 and 221.1 kJ mol^−1^ under the CF reaction to 64.8 and 32.9 kJ mol^−1^ under the MW reaction for zinc oxide and zinc ferrite reduction, respectively.

In summary, the enhancements in the zinc reduction and recovery rates not only resulted from the rapid and bulk heating of the MW reaction, but also from the MW non-thermal effects related to the interactions occurring between the microwave electromagnetic field and the reactant molecules.

## Conflicts of interest

There are no conflicts to declare.

## Supplementary Material

RA-010-D0RA04574H-s001
