# Supplementary material for: Microwave catalyzed carbothermic reduction of zinc oxide and zinc ferrite: effect of microwave energy on the reaction activation energy
Source: RSC Adv. 2020 Jun 23;10(40):23959–68. doi: 10.1039/d0ra04574h (PMC9055094; doi:10.1039/d0ra04574h)
Supplement: RA-010-D0RA04574H-s001 [file RA-010-D0RA04574H-s001.pdf]

Electronic Supplementary Material (ESI) for RSC Advances.
This journal is © The Royal Society of Chemistry 2020

Supplementary Material

For

Microwave Catalyze Carbothermic Reduction of Zinc Oxide and Zinc Ferrite: Effect of Microwave Energy on the Reaction Activation Energy

Mamdouh Omran[a,b*], Timo Fabritius[a], , Eetu-Pekka Heikkinen[a], Tero Vuolio[a], Yaowei Yu[b], Guo Chen[c**], Yilmaz Kacar[d]

[a] Process Metallurgy Research Group, Faculty of Technology, University of Oulu, Oulu, Finland
[b] State Key Laboratory of Advanced Special Steel, School of Materials Science and Engineering, Shanghai University
[c] Kunming Key Laboratory of Energy Materials Chemistry, Yunnan Minzu University, Kunming 650500, P.R. China.
[d] Material science and engineering Dep., Carnegie Mellon University, Pittsburgh, PA 15213, USA.

[*]Corresponding author:

Mamdouh Omran

Address: Process Metallurgy Research Group, Faculty of Technology, University of Oulu, Finland. P.O. Box: 4300.

E-mail: mamdouh.omran@oulu.fi mamdouh.omran@oulu.fi

Co-corresponding author:

Guo Chen

E-mail: guochen@kust.edu.cn

I. Dielectric properties

The microwave-absorbing ability of materials primarily depends on their complex permittivity (ε) (Lovas et al., 2010; Li et al., 2020). Complex permittivity ε is expressed as Eq. 1:

$$\varepsilon = \varepsilon' - j\varepsilon'' \quad (1)$$

where ε' is the real part of the complex permittivity, ε" is the imaginary part of the complex permittivity, and $j = (-1)^{1/2}$ (2)

The real permittivity (ε′) is called the dielectric constant, and the imaginary permittivity (ε″) is called the dielectric loss factor. ε′ indicates the ability of the material to absorb electromagnetic radiation within its structure, whereas ε″ represents the ability of a material to dissipate the adsorbed radiation into heat (Li et al., 2019; Li et al., 2020).

The ratio of the loss factor to the dielectric constant is known as the loss tangent (tan δ_d). The loss tangent (tan δ_d) is an important factor that provides an indication of how well a material dissipates stored energy into heat (Li et al., 2019; Li et al., 2020). The loss tangent is expressed as Eq. 3:

$$tan\delta_d = \frac{\varepsilon''}{\varepsilon'} \quad (3)$$

In this work, the dielectric properties of the studied samples were measured using an open-ended coaxial probe at frequencies of 1064 MHz and 2423 MHz and temperatures between 25 to 1100 °C. The details of this procedure are described in previous work (Omran et al., 2019).

References:

Li, K., Chen, G., Li, X., Peng, J., Ruan, R., Omran, M., Chen, J., 2019. High-temperature dielectric properties and pyrolysis reduction characteristics of different biomass-pyrolusite mixtures in microwave field. Bioresour. Technol. 294, 122217.

Li, K., Chen, J., Peng, J., Ruan, R., Omran, M., Chen, G., 2020. Dielectric properties and thermal behavior of electrolytic manganese anode mud in microwave field. J. Hazard. Mater.384, 121227.

Lovas, M., Kovacova, M., Dimitrakis, G., Čuvanova, S., Znamenaćkova, I., Jakabsky, Š., 2010. Modeling of microwave heating of andesite and minerals. Int. J. Heat Mass. Transf. 53, 3387-3393.

Omran, M., Fabritius, T., Chen, G., He, A., 2019. Microwave absorption properties of steelmaking dusts: effects of temperature on the dielectric constant (ε') and loss factor (ε") at 1064 MHz and 2423 MHz, RSC Adv. 9, 6859-6870.

Omran, M., Fabritius, T., Elmahdy, A., Abdel-Khalek, N.A., El-Aref, M., Elmanawi, A., 2014. Effect of microwave pretreatment on the magnetic properties of iron ore and its implications on magnetic separation. Sep. Purif. Technol.

136, *223-232*.

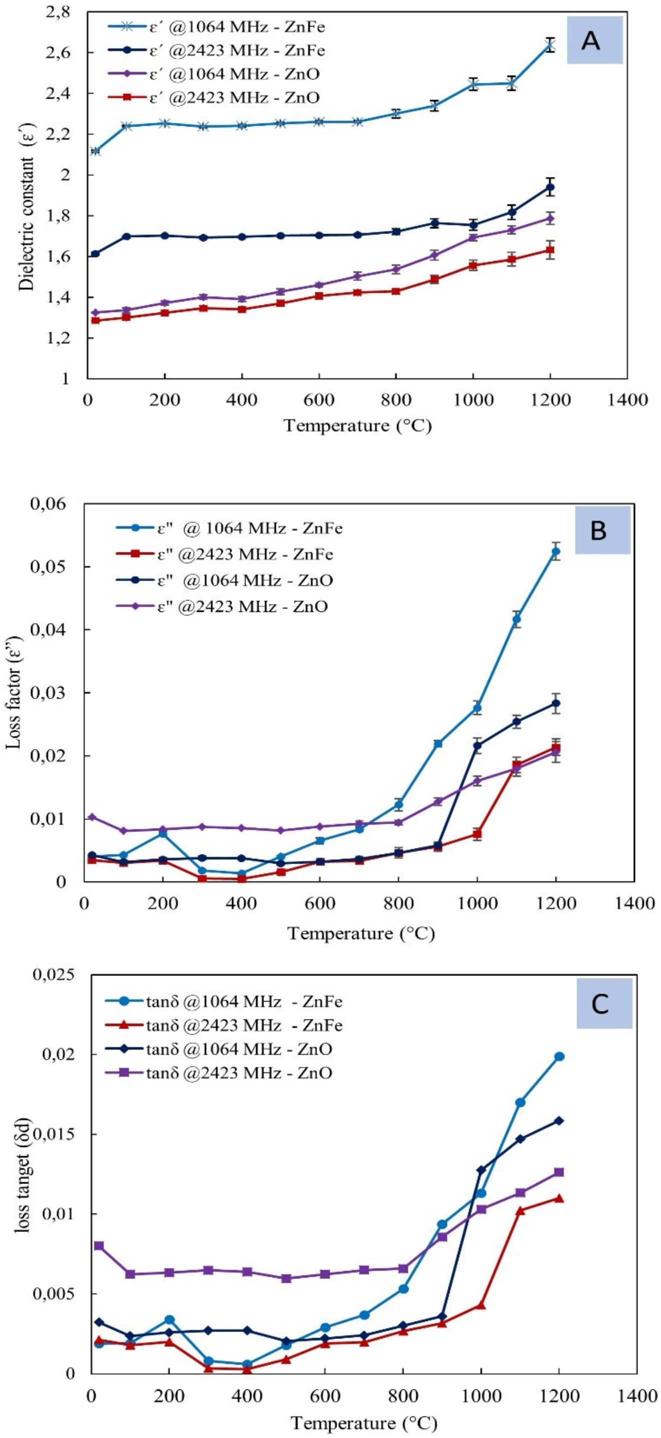

Fig. S1. Dielectric properties of zinc oxide and zinc ferrite at frequencies of 1064 MHz and 2.423 GHz. (A) dielectric constant (ε'); (B) loss factor (ε''); (C) loss tangent (δ_d).

The ε' of ZnO and $ZnFe_2O_4$ at temperatures between 20 and 1200 °C at 1064 MHz and 2423 MHz frequencies are presented in Fig. (S1). The changes in ε' with temperature were insignificant. Fig. (S1A) shows that ε' stays almost constant within the temperature range of 100 to 100 °C. There was a slight increase toward the high end of the temperature range. The ε'' of ZnO and $ZnFe_2O_4$ at temperatures between 20 and 1200 °C and at 1064 MHz and 2423 MHz frequencies are presented in Fig. (S1B). Changes in ε'' are more significant at higher temperatures. The ε'' stays approximately constant until reaching a temperature of 800 °C (a small increase in ε'' values). When temperature exceeded 900 °C, a sharp increase in the ε'' values was observed. At a frequency of 1064 MHz, ε'' increased from 0.0051 to 0.027 and from 0.02 to 0.08 for ZnO and $ZnFe_2O_4$, respectively. Based on the comparison with the DSC curve, the greater changes in ε'' appear at temperatures when the sample began sintering. The increase in ε'' at higher temperatures can be attributed to an increase in electrical conductivity due to sample sintering.

Fig. S1C shows the loss tangent of ZnO and $ZnFe_2O_4$ as a function of temperature at frequencies of 1064 MHz and 2423 MHz. It can be seen that the behavior of the loss tangent for both ZnO and $ZnFe_2O_4$ with temperature coincides with the loss factor (ε'') behavior.

II. Isothermal reduction experiments

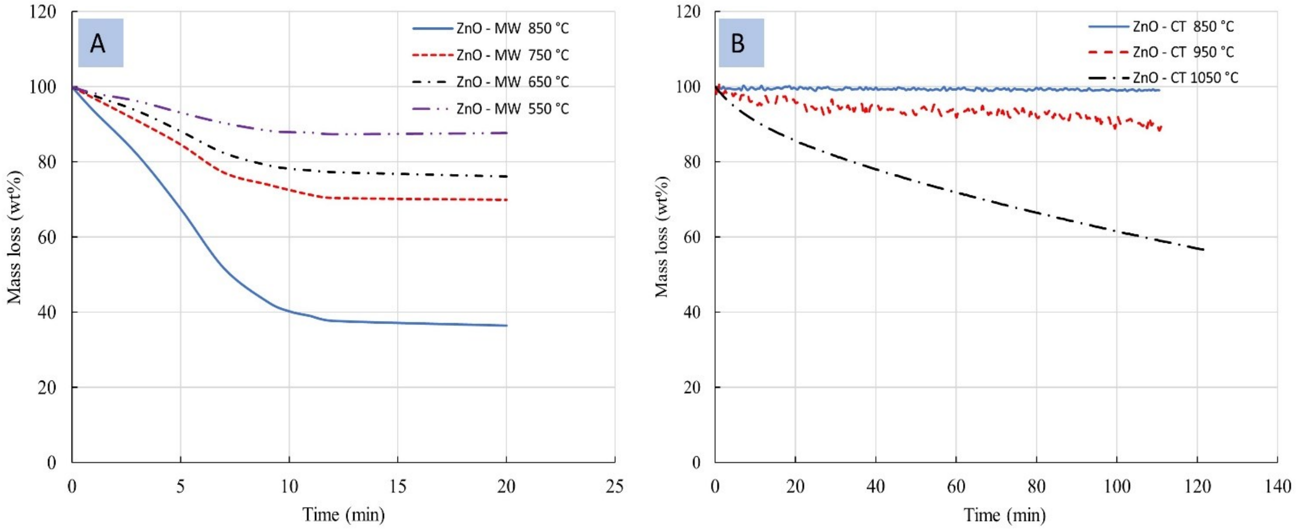

Fig. S2. Experimental mass loss for zinc oxide. (A) MW reaction; (B) CT reaction.

The sample mass loss during the carbothermic reduction of zinc oxide from the MW and CT reactions as a function of temperature is shown in Fig. S2A and S2B, respectively.

Under MW, we observed a rapid mass loss in the early stages of the reaction; however, after approximately 11 min, the mass became constant. In the case of the MW reaction, a mass loss of about 11.87 wt.% was obtained at 550 °C, indicating the reduction of ZnO initiated at this temperature. The mass loss increased as the temperature increased. Under the CT reaction (Fig. S2B), the sample mass was almost unchanged at 850 °C, indicating that the reduction of ZnO was not favorable at this temperature. By increasing the temperature to 950 °C, a mass loss of 11.63 wt.% was observed, indicating that the reduction of ZnO initiated at this temperature, which was consistent with the thermodynamic equilibrium calculations. The mass loss increased steadily as a function of temperature, and the mass loss under the CT conditions was quite linear with time.

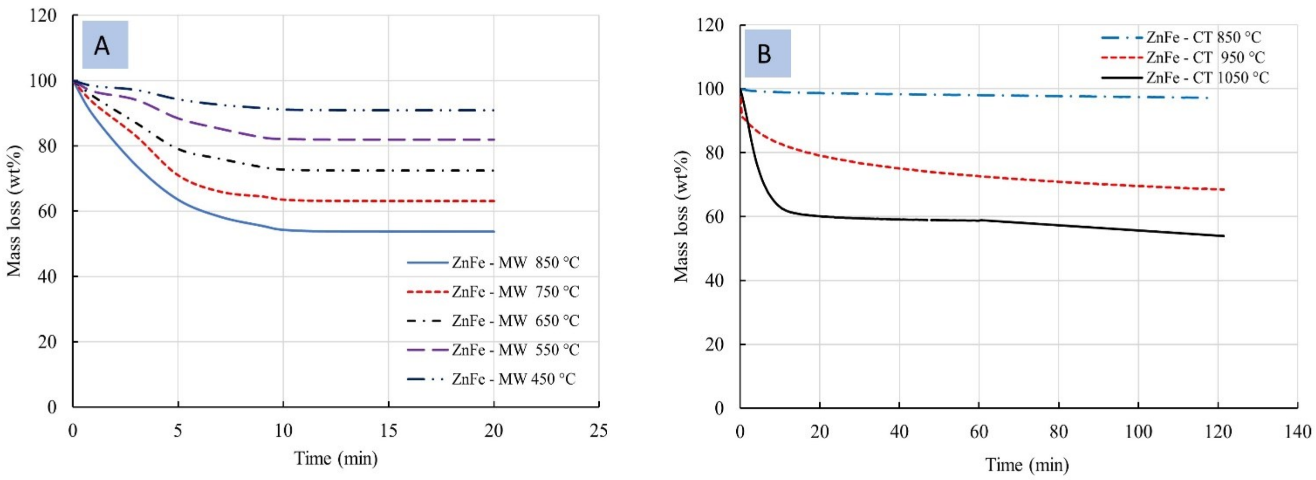

Fig. S3. Experimental mass loss for zinc ferrite. (A) MW reaction; (B) CT reaction.

Fig. (S3) shows the sample mass loss of zinc ferrite under the MW and CT reactions as a function of temperature.

Under the MW reduction (Fig. S3A), there was a rapid mass loss in the early stages of the reaction; after that, the sample mass became constant. Under the CT reduction (Fig. S3B), we also observed a rapid mass loss in the very early stages of the reaction, after which the mass loss became gradual. The rapid mass loss for zinc ferrite was owing to its high reactivity compared to zinc oxide. In the case of the CT reaction, we did not observe any mass loss in temperature lower than 850 °C. About ~ 3 wt.% mass loss was observed at 850 °C, revealing that zinc ferrite decomposition was initiated at this temperature. By increasing the temperature, the mas loss increased. Meanwhile, in the case of the MW reaction, a mass loss of ~ 9.91 wt.% obtained at 450 °C indicated that zinc ferrite decomposed at about 450 °C. Nearly ~54 wt.% mass loss was obtained for both the MW and CT reactions at temperatures of 850 °C and 1050 °C, respectively.
